# Stability of Oxytocin Preparations in Malawi and Rwanda: Stabilizing Effect of Chlorobutanol

**DOI:** 10.4269/ajtmh.20-0255

**Published:** 2020-08-03

**Authors:** Nhomsai Hagen, Thomas Bizimana, Pierre Claver Kayumba, Felix Khuluza, Lutz Heide

**Affiliations:** 1 Pharmaceutical Institute, Eberhard Karls University Tübingen, Tübingen, Germany;; 2 School of Medicine and Pharmacy, College of Medicine and Health Sciences, University of Rwanda, Kigali, Rwanda;; 3 East African Community Regional Centre of Excellence for Vaccines, Immunizations and Health Supply Chain Management (EAC RCE-VIHSCM), University of Rwanda, Kigali, Rwanda;; 4 Pharmacy Department, College of Medicine, University of Malawi, Blantyre, Malawi

## Abstract

Oxytocin is used for the prevention and treatment of postpartum hemorrhage, the leading cause of maternal mortality in low- and middle-income countries. Because of the high instability of oxytocin, most products are labeled for storage at 2–8°C. Some other products are on the market which are labeled for non-refrigerated storage, but independent evaluations of their stability hardly exist. In the present study, seven brands (nine batches) of oxytocin were purchased from wholesalers and medical stores in Malawi and Rwanda and investigated by accelerated stability testing according to the ICH/WHO guidelines. Two oxytocin brands approved by a stringent regulatory authority (SRA) or by the WHO Prequalification of Medicines program and purchased in Europe were used as comparison. All investigated brands which were either produced in countries with SRAs, or were WHO-prequalified products, were labeled for storage at 2–8°C, and all of them passed stability testing with very good results. Even exposure to 25°C or 30°C for several months hardly affected their oxytocin content. However, two other investigated brands were labeled for non-refrigerated storage, and both of them had been produced in countries without SRAs. These two preparations showed not higher but lower stability than the brands labeled for storage at 2–8°C, and, for both of them, noncompliance with pharmacopoeial specifications was found after accelerated stability testing. At 40°C, and in forced degradation studies at 80°C, chlorobutanol showed a remarkable stabilizing effect on oxytocin, which may deserve further investigation. The results of the present study support the policy “Buy Quality Oxytocin, Keep It Cool.”

## INTRODUCTION

Oxytocin is listed in the essential medicines list of the WHO^1^ and is used for the prevention and treatment of postpartum hemorrhage (PPH).^2^ Postpartum hemorrhage is the leading cause of maternal mortality in low-income countries.^2^ One of the sustainable development goals of the United Nations is “to reduce the global maternal mortality ratio to less than 70 per 100,000 live births by 2030.”^3^ To achieve this, prevention and treatment of PPH with oxytocin is an essential step.

**Figure 1.**
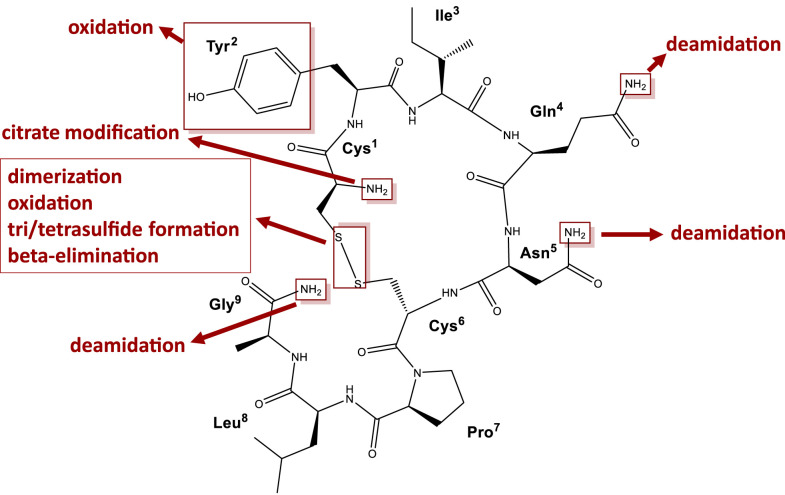
Structure of oxytocin and its typical degradation mechanisms. Modified from Avanti et al.^39^ This figure appears in color at www.ajtmh.org.

Several previous studies have shown that the quality of oxytocin, especially in low- and middle-income countries (LMICs), is often poor.^4–13^ This may result from poor manufacturing (e.g., inappropriate formulation or packaging), from poor storage and/or transportation conditions, or from a combination of these factors. The nonapeptide oxytocin is known to be very sensitive to high temperatures.^4,14–17^ The typical degradation mechanisms are shown in Figure 1. If oxytocin is not stored properly, then it may lose its potency, which can result in higher mortality rates of PPH.

Most of the currently marketed oxytocin products have to be stored at 2–8°C according to the labeling information. Maintaining this storage temperature can present a challenge, especially in rural health facilities in LMICs.^6,9,13,18,19^ Several oxytocin preparations are now on the market which are labeled for non-refrigerated storage. However, according to a report of the Reproductive Health Supplies Commission, there are nearly 300 different oxytocin products, offered by at least 100 manufacturers, and there is considerable confusion about the storage and labeling requirements for these.^20^ Similar concerns were raised by the WHO in a report on the quality of medicines for maternal and child health, which concluded for oxytocin injections: “It would be useful to verify to which extent manufacturers’ instructions for higher storage temperatures were based on reliable stability studies.”^11^ The Promoting the Quality of Medicine Program of the U.S. Pharmacopeia stated a need to conduct “systematic stability studies […] in accordance with the ICH guidelines […], to reassess the specifications for storage and shelf life of oxytocin injections.”^21^ It was with these recommendations in mind that we decided to investigate the stability of selected oxytocin preparation according to the ICH guidelines (see Methods).

The stability of pharmaceutical products must be investigated and documented by the manufacturers, and National Medicines Regulatory Authorities (NMRAs) usually do not confirm these data independently. Countries with the so-called stringent regulatory authorities (SRAs) are usually trusted to ensure complete and correct stability testing by the manufacturers. Stringent regulatory authorities are NMRAs who are members, observers, or associates of the International Council of Harmonization of Technical Requirements for Registration of Pharmaceuticals for Human Use (ICH) and currently comprise the national regulatory authorities of the EU member states, the United States, Japan, Switzerland, Canada, Australia, Iceland, Liechtenstein, and Norway.^22^ In addition, the WHO prequalification of medicines program (now called “WHO Prequalification Team: medicines”) prequalifies finished pharmaceutical products for certain indications, including reproductive and maternal health, based on information submitted by manufacturers and on inspections of the corresponding manufacturing sites.^23,24^

However, many medicines circulating in LMICs are neither approved by a stringent regulatory authority (SRA) nor WHO prequalified. National regulatory authorities of LMICs often have only limited capacity for independent evaluation and analysis^25,26^ and need to rely on manufacturers’ data when registering medical products. Independent scientific confirmations of manufacturers’ claims, for example, on heat-stability of oxytocin preparations, are therefore important.

The ICH has formulated guidelines for stability testing of pharmaceutical products (Q1A-Q1F).^27^ Testing a product’s stability over its entire shelf life is very time consuming; therefore, accelerated stability studies for a shorter time, but at higher temperature and relative humidity (RH), are permissible and are frequently used for the prediction of shelf life and storage requirements. The precise conditions for accelerated stability studies depend on the intended storage recommendation, as well as on the climatic zone of the country where the medicine will be registered, as specified by the ICH^28^ and by the WHO^22^ guidelines. Malawi is currently assigned to WHO climatic zone II (subtropical and Mediterranean) and Rwanda to zone IVa (hot and humid).^29^

The so-called forced degradation studies^30^ are carried out at even higher temperatures. They were used in the current study to investigate the influence of excipients such as buffers and the widely used bacteriostatic agent chlorobutanol on the stability of oxytocin.

## MATERIALS AND METHODS

### Study design and ethical approval.

This study followed the WHO guidelines on the conduct of surveys of the quality of medicines^31^ and the MEDQUARG guidelines,^32^ where applicable. Ethical clearance to conduct this study was received from the College of Medicine Research and Ethics Committee in Malawi (Reference no. P.07/27/2215). Approval was also granted by the Malawi National Regulatory Agency (Pharmacy, Medicines and Poisons Board [PMPB]), as well as by the Ministry of Health, Rwanda (Reference no. 20/1361/DGPHFIS/2018).

### Sample collection.

In Malawi and Rwanda, all brands of oxytocin available during the time of sample collection (February–March 2018) at government and faith-based medical stores (i.e., at the Central Medical Stores Trust in Malawi, the Rwanda Biomedical Center/Medical Production, Procurement and Distribution Division of Rwanda, and the Bureau des Formations Médicales Agréées du Rwanda in Rwanda) and from all pharmaceutical wholesalers located in Blantyre and Lilongwe (Malawi) and in Kigali (Rwanda) were purchased by local researchers (F. K. and T. B.). For comparison, additional samples were purchased from the pharmacy of the university hospital in Tübingen, Germany, and from an international wholesaler (Imres B.V., Lelystad, The Netherlands). A mystery shopper approach was used when ordering from commercial wholesalers. An overt approach was used when samples were collected in person from government or faith-based medical stores. For each brand, 100 vials of oxytocin were purchased. If different batches of a certain brand were available, then samples from each batch were purchased. After purchase, samples were stored according to the manufacturers’ specifications, and temperature loggers were kept with all samples until their placement in stability chambers. Samples were hand-carried by the investigators from Malawi or Rwanda to Germany via airplane, with traveling time of less than 24 hours. At the Pharmaceutical Institute of Tübingen University, samples were pretested for assay and pH according to the U.S. Pharmacopeia. All samples that were within specifications at the time of pretesting were included into the stability study.

### Storage in stability chambers.

Samples were stored in four different stability chambers (Table 1) for 6 months (April–October 2018) at Alpha-Pharma-Service GmbH, Heilbronn, Germany. Conditions were chosen in accordance with the ICH and WHO guidelines for stability testing of pharmaceutical products.^22,28^ At different time points (months 0, 1, 2, 3, and 6), samples were withdrawn from the chambers and analyzed at the Pharmaceutical Institute at Tübingen University, Germany.

**Table 1:** Conditions for accelerated stability testing and controls

Temperature (°C)	Relative humidity (%)	ICH/WHO* stability testing conditions	
		For refrigerated products	For non-refrigerated products
5 ± 3	n. def.	Long term	–
25 ± 2	60 ± 5	Accelerated	Long term, zone II
30 ± 2	65 ± 5	Accelerated, more severe*	Long term*, zone IVa, intermediate
40 ± 2	75 ± 5	–	Accelerated

n. def. = not defined. According to the ICH/WHO,^22,28^ long-term testing should cover the proposed shelf life or a minimum of 12 months at the time of submission. Stability testing under accelerated or intermediate conditions should cover a minimum of 6 months.

* In addition to the conditions listed in the ICH guidelines, the WHO guidelines list also more severe conditions for products that are intended to be marketed in climatic zones III–IV. Malawi is assigned to climatic zone II and Rwanda to climatic zone IVa.^29^

### Sample analysis.

At the Pharmaceutical Institute of Tübingen University, Germany, samples were visually inspected, and tested for identity, assay, and pH value according to the monograph of the U.S. Pharmacopoeia (USP 40, oxytocin injections). Methods were validated according to USP 40. Analysis was conducted via high-performance liquid chromatography (HPLC, Agilent Infinity 1260 II with a binary pump, a variable wavelength detector, a refrigerated autosampler, and an integrated column compartment; Agilent Technologies, Santa Clara, CA). Solvents were HPLC grade.

For analysis, a gradient system of mobile phase A (0.1 M NaH_2_PO_4_ buffer) and mobile phase B (ACN: H_2_0 1:1 V/V) with the following gradient was used: 0 minutes, 30% B; 10 minutes, 40% B; 17.5 minutes, 65% B; 20.5 minutes, 65% B; 23.5 minutes, 30% B; 26 minutes, 30% B; flow rate, 1.5 mL/minute; injection volume, 70 µL; column with guard from Dr. Maisch GmbH, Ammerbuch, Germany (Reprospher 100: 12.5 cm × 4.6 mm, 5 µm C18); and detection at 220 nm. The U.S. Pharmacopoeia Reference Standard (batch N° F3K133) was obtained from Merck KGaA, Darmstadt, Germany. pH values were tested with a pH-combination microelectrode (N 6000 BNC) from SI Analytics GmbH, Mainz, Germany. From each sample, three vials were analyzed.

Five-point calibration curves were prepared at each time point (i.e., months 0, 1, 2, 3, and 6) to assure linearity. Intermediate precision^33^ was calculated from the data of the calibration curves of the reference standards, which showed relative SDs (RSDs) of less than 2.1% for the reference solution corresponding to 100% of the declared content (Supplemental Table S3).

### Forced degradation study.

Both the ICH and WHO guidelines mention forced degradation studies (sometimes referred to as stress testing); however, they do not fix specific conditions under which forced degradation studies should be performed. In the present study, the conditions described by Blessy et al.^30^ in 2013 were used. The commercial preparations of oxytocin were investigated in their original glass vials, and the commercial preparation of oxytocin from Hexal with added chlorobutanol (5 and 1.5 mg/mL) was investigated in tightly sealed flasks. Furthermore, solutions prepared from solid synthetic oxytocin (Sigma-Aldrich/Merck, Taufkirchen, Germany; Batch N° 103H05241; purity ≥ 97% by HPLC), in a concentration of 10 IU/mL in either distilled water or 0.2 M sodium acetate buffer pH 4.6, both either with or without addition of chlorobutanol (1.5 or 5 mg/mL), were prepared and investigated. All samples were stored for 5 days at 80°C. After 24, 72, and 120 hours, one sample of each formulation was removed from the heat chamber and stored at 4°C until analysis. For each formulation, a sample stored at 4°C was used as control. Analysis was carried out as described previously.

### Registration status of medicine brands.

The PMPB of Malawi was contacted to inquire the registration status of the medicines collected in Malawi. For the preparations by Ningbo Pharma Biotech Co. Ltd., Ningbo, Zhejiang, China, Umedica Laboratories Pvt. Ltd., Mumbai, India, and Ciron Drugs & Pharmaceuticals Pvt. Ltd., Mumbai, India, it was confirmed that they were registered, but registration could not be confirmed for the product by Biologici Italia Laboratories S.r.l., Masate, Italy. The Rwanda Food and Drug Authority (RFDA) was contacted to inquire the registration status of the medicines collected in Rwanda. However, the RFDA’s process of full registration of medicines had not yet been in effect at the time of sample collection. It could not be confirmed which of the collected preparations were preregistered according to the previous procedures.

The preparations of Biologici Italia Laboratorie S.r.l., of Rotexmedica GmbH, Trittau, Germany and of AS Grindeks, Riga, Latvia, were also registered in European countries, as was the preparation of Hexal AG, Holzkirchen, Germany. However, no oxytocin preparation by Laboratoires Sterop, Brussels, Belgium, was found in the medicine registers of the European Heads of Medicines Agencies (https://mri.cts-mrp.eu/Human/) and in the national medicines register of Belgium (https://banquededonneesmedicaments.afmps-fagg.be/#/query/human/), indicating that the Sterop preparations collected in Rwanda are manufactured for export only.

### Statistical analysis.

Statistical evaluation was carried out using JMP 14.2 (SAS GmbH, Heidelberg, Germany). Significance of differences between active pharmaceutical ingredient (API) content and pH values at different conditions and months were calculated using uni- and multivariate analysis of variance and Student’s *t*-test.

### Information of national authorities and stakeholders.

This article was shared with the PMPB of Malawi, with the RFDA, and with the WHO Rapid Alert System. In addition, the results were presented to the PMPB, the Malawi Central Medical Stores Trust, the Ministry of Health and national and international stakeholders during a meeting in Lilongwe, Malawi, on September 4, 2019. First results of this study were also presented in a lecture by L. H. during a visit to the RFDA on December 4, 2018.

## RESULTS

### Overview of investigated oxytocin samples.

Nine brands of oxytocin, that is, five brands in Malawi and four in Rwanda, were collected. Two of the purchased brands had to be excluded from the subsequent investigation: oxytocin inj. 5 IU/mL from MACIN Remedies Ltd., Moga, India, collected in Malawi, was not available in a sufficient amount for stability testing. Oxytocin inj. 10 IU/mL from Jiangxi Xierkangtai Pharmaceutical Co. Ltd., Pingxiang, Jiangxi, China, collected in Rwanda, showed a large peak of an undeclared substance (later identified as the commonly used preservative benzyl alcohol^34^) in HPLC analysis, which precluded precise quantitative analysis of oxytocin because of lack of baseline separation of the closely adjacent HPLC peaks of benzyl alcohol and oxytocin. Therefore, seven brands collected in Malawi and Rwanda were included into the subsequent stability testing. For two of these brands, two different batches were offered at the time of collection, and, in each case, both batches were collected and investigated. As a comparison, two further oxytocin preparations were purchased in Europe: one preparation which is commonly used in Germany, purchased through the pharmacy of the University Hospital Tübingen, and one of the two oxytocin preparations which had been prequalified by the WHO at the time of sample collection; the latter preparation was purchased from the manufacturer in Latvia via Imres B.V. because it was not registered in Germany. A different batch of the latter brand had also been purchased in Rwanda.

Therefore, as shown in Table 2, eight brands (total 11 batches) were included into the stability testing. Four of these brands had been produced in Belgium, Germany, or Italy, that is, in countries with an SRA.^22^ As mentioned, one further brand was produced in Latvia and represented a WHO-prequalified product.^35^ The other three brands were from India and China, that is, from countries without an SRA. Six of the eight brands were labeled for storage at 2–8°C, whereas two brands, produced in China and India, were labeled for storage “below 25°C” and “not exceeding 30°C,” respectively (Table 2). The declared shelf life of the different products varied between 2 and 4 years, and all samples remained within their shelf life during the entire duration of the study. Pretesting for assay and pH showed that 10 of the 11 samples were clearly within USP 40 specifications at the beginning of the stability study. One sample (Ningbo batch no. 160802) showed an assay value of 89.0%. Given the RSD of this measurement (RSD = 1.41%; Supplemental Table S1), this deviation from the pharmacopoeial limits (90–110%) was not statistically significant (*P* = 0.236); therefore, this preparation was not classified as being out of specifications.

**Table 2:** Investigated oxytocin samples

Collected in	Stated manufacturer (and brand name, if not marketed under INN)	Country of manufacture	Expiry date	Batch number	Stated shelf life (years)	Stated storage requirements (°C)	Stated (or detected) excipients								Prequalification status
							Water for inj.	Chlorobutanol (mg/mL)	Sodium acetate	Acetic acid	Sodium hydroxide	Sodium chloride	Citric acid	Others	
Malawi	Ningbo Pharma Biotech Co., Ltd. (WW-Oxy 10)	China	August 19	160802	3	Less than 25	**+**	–	–	–	–	–	–	+*	None
			January 19	160183											
	Umedica Laboratories Pvt. Ltd.	India	January 20	JA802	2	Not exceeding 30	**+**	1.5†	–	–	–	–	–	+*	None
	Ciron Drugs & Pharmaceuticals Pvt. Ltd.	India	August 19	7EA01228	2	2–8	**+**‡	5†	–	–	–	–	–	–	None
	Biologici Italia Laboratories S.r.l.	Italy	March 19	UF602ON	3§	2–8	**+**	–	**+**	**+**	**+**	–	–	–	SRA
Rwanda	Laboratoires STEROP	Belgium	August 19	160269	3	2–8	**+**	5	–	–	–	**+**	**+**	–	SRA
	Laboratoires STEROP (Steroxine 10 IU/1 mL)‖		January 19	160042											
	Rotexmedica GmbH Arzneimittelwerk	Germany	September 20	70779A	3	2–8	**+**	–	**+**	**+**	–	**+**	–	–	SRA
	AS Grindeks	Latvia	November 20	37711116	4	2–8	**+**	–	**+**	**+**	**+**	**+**	–	–	WHO-PQ
Europe	AS Grindeks		September 21	38910917											
	Hexal AG	Germany	June 20	HC0075	3¶	2–8	**+**	–	–	**+**	–	**+**	–	–	SRA

WHO-PQ = WHO-prequalified product; SRA = produced in a country with stringent regulatory authority. All samples represented 1 mL vials with a stated content of 10 IU oxytocin/mL.

* High-performance liquid chromatography (HPLC) analysis showed additional peaks, indicating additional, unidentified ingredients.

† Chlorobutanol not declared but detected in HPLC analysis.

‡ Water for inj. not explicitly declared.

§ Manufacturing date not stated on the packaging; information received from the websites of the British Medicines and Healthcare products Regulatory Agency (http://www.mhra.gov.uk) and the Irish Health Products Regulatory Authority (http://www.hpra.ie/).

‖ One branded and one unbranded generic oxytocin product of Laboratoires STEROP were found in Rwanda, with identical composition.

¶ Manufacturing date not stated on the packaging but received from the manufacturer.

Table 2 also shows the stated (or detected) excipients in the investigated preparations. For optimal stability of oxytocin, USP 40 specifies that the pH value must be between 3.0 and 5.0. This is usually achieved by the inclusion of, for example, acetate or citrate buffers.^36–39^ For the WHO-prequalified preparation, and for the four preparations produced in countries with an SRA, the presence of such buffering substances was correctly declared on the packaging. According to the label information, four of these five preparations also contained sodium chloride (usually included for tonicity). For the Sterop preparation, in addition, the presence of 5 mg/mL of the bacteriostatic agent chlorobutanol was declared.

In clear contrast, for the three preparations from China and India, no excipients were stated on their packaging beyond water for injection (for the Ciron preparation, not even water was stated). Nevertheless, their pH value was found to be in the correct range of 3.0–5.0, and remained so during stability testing, which may indicate the presence of undeclared buffering agents. Furthermore, HPLC analysis showed the (undeclared) presence of 1.5 and 5.0 mg/mL chlorobutanol in the preparations by Umedica and Ciron, respectively. Chlorobutanol can be readily detected in the HPLC analysis.

### Accelerated stability studies of oxytocin.

All eight brands (11 batches) listed in Table 2 were subjected to accelerated stability testing according to the current WHO guidelines for stability testing of finished pharmaceutical products.^22^ Stability chambers used, operated by a commercial company specialized in pharmaceutical stability testing, also complied with the specifications for RH of the WHO guidelines, although this is not relevant for the investigated oxytocin preparations which were packaged as aqueous solutions in sealed glass vials which are considered to be moisture impermeable.^22^

The evaluation criteria for stability testing are described in full detail in the mentioned WHO guidelines,^22^ but may be summarized as follows: pass/fail decisions of accelerated stability testing are made on the basis of two criteria: 1) for 6 months, the sample must remain within specification, that is, in the present study, oxytocin samples still had to show an API content between 90% and 110% of the declared amount (as specified by the USP, International Pharmacopeia, and British Pharmacopeia), and a pH value between 3.0 and 5.0; 2) no significant change of the API content must occur within 6 months, that is, no change of the initial content of the API by 5% or more.^22,28^

Obviously, the temperature conditions which the oxytocin preparations must be able to withstand in accelerated stability testing are related to the storage recommendations which the manufacturer states on the label^22^: products labeled for refrigerated storage (2–8°C) must demonstrate their stability for 6 months at 25°C or 30°C, and the decision for either of these two temperatures should be “based on a risk-based evaluation.” As described in the following text, in the present study, results obtained at 25°C and at 30°C were similar. Products labeled as “do not store above 25°C,” such as the oxytocin preparation by Ningbo (Table 2), must demonstrate their stability for 6 months at 30°C (“intermediate” condition, tested in case they failed at 40°C). And products labeled as “do not store above 30°C,” such as the oxytocin preparation by Umedica (Table 2), must demonstrate their stability for 6 months at 40°C.

**Figure 2.**
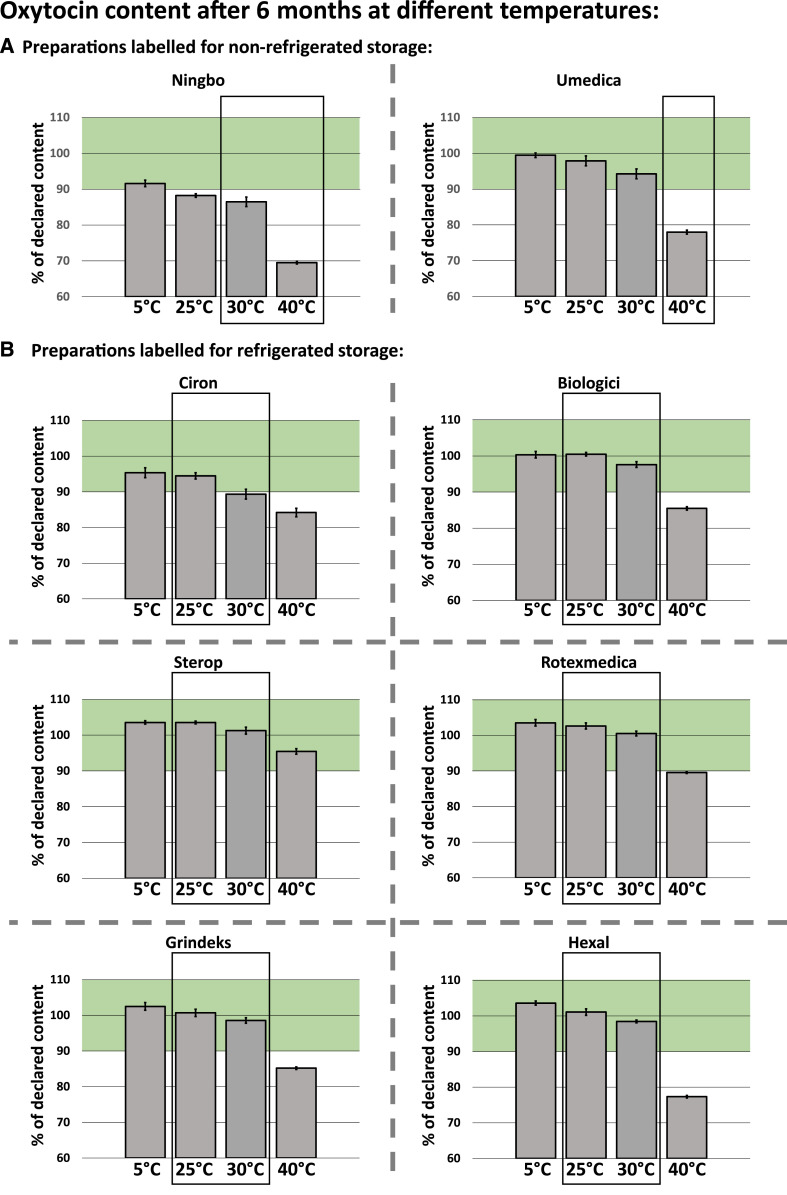
Accelerated stability testing of commercial oxytocin preparations: oxytocin content after 6 months at different temperatures. Full names of the manufacturers, and further details of the investigated preparations, are listed in Table 2. Results shown here were obtained with Ningbo batch N° 160802, Sterop batch N° 160269, and Grindeks batch N° 37711116 (see Table 2). The U.S. Pharmacopoeia specifies that the oxytocin content must be between 90% and 110% of the declared amount. This range is marked in the diagrams. Error bars show SD. This figure appears in color at www.ajtmh.org.

Figure 2 shows the oxytocin content of all eight brands listed in Table 2 after 6 months of storage at 5°C, 25°C, 30°C, or 40°C. Clearly, the WHO-prequalified preparation as well as the four preparations produced in countries with an SRA passed this stability test, showing oxytocin contents between 97.4% and 109.3% of the declared amount after 6 months at 30°C. Supplemental Table S1 lists the individual assay values determined for each preparation at each time interval (0, 1, 2, 3, and 6 months); none of the mentioned five brands showed a significant change of the API content over 6 months at 30°C; that is, all passed the stability testing also by this criterion. Supplemental Table S2 shows all pH values determined in this study. In all five mentioned preparations, the pH value remained well within specifications, that is, within the 3.0–5.0 range at 30°C.

For the preparation by Ciron, an API content of 89.3% of the declared amount was determined after 6 months at 30°C. Given the RSD of this measurement (RSD = 1.55%, Supplemental Table S1), this deviation from the pharmacopoeial limits (90–110%) is not statistically significant (*P* = 0.459); therefore, this preparation was not classified as failing stability testing. The change of the API content of this preparation, and its pH value, was within the permitted limits.

**Figure 3.**
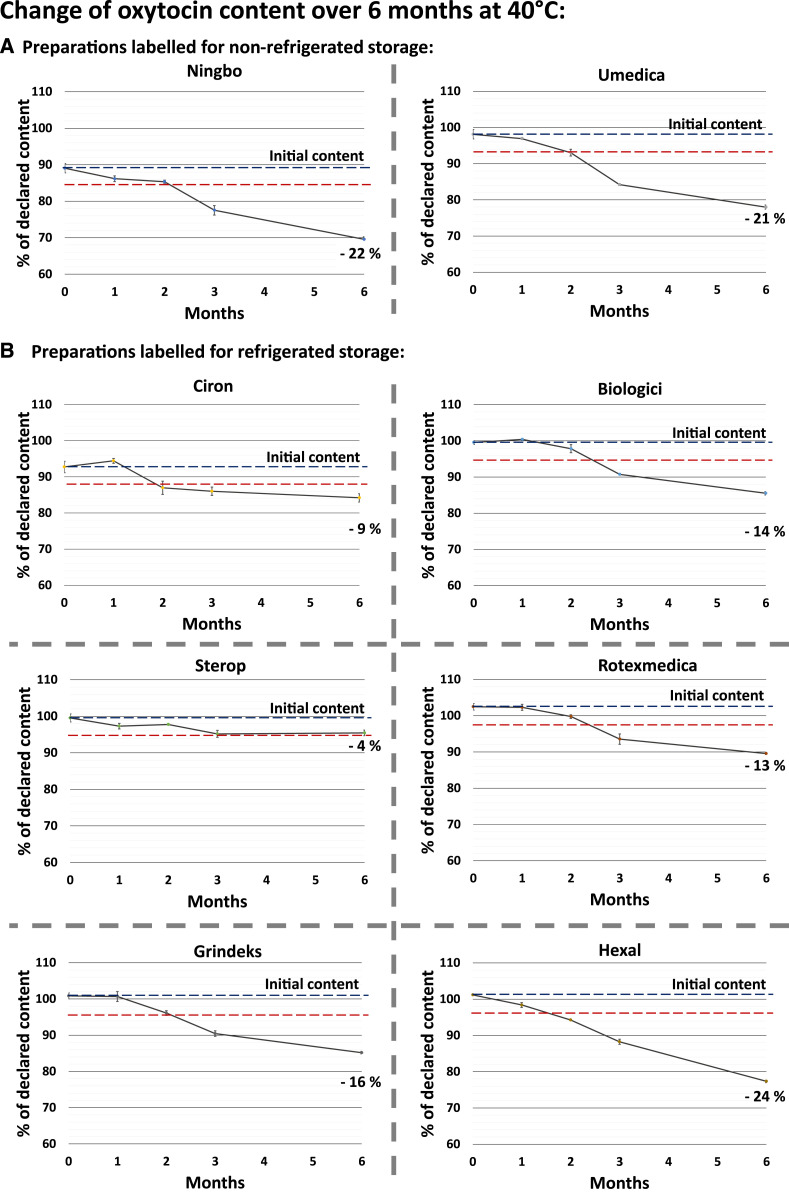
Accelerated stability testing of commercial oxytocin preparations: change of oxytocin content over 6 months at 40°C. Full names of the manufacturers, and further details of the preparations, are listed in Table 2. Results shown here were obtained with the same batches as shown in Fig. 2. In stability testing, a “significant change” of the API content is defined as a 5% change of the initial content.^22,28^ Initial content and initial content minus 5% are shown as dashed lines. Error bars show SD. Final loss of content after 6 months is calculated relative to the initial content. This figure appears in color at www.ajtmh.org.

A different picture emerged for the preparations by Ningbo and Umedica, that is, for the two preparations in this study which were labeled by their manufacturers as not requiring refrigerated storage. As immediately obvious from Figure 2, their stability at 30°C and 40°C was not better but rather lower than that of the preparations labeled for refrigerated storage. The product by Umedica was labeled for storage “not exceeding 30°C” and, by WHO guidelines, therefore had to remain within specifications for 6 months at 40°C. It clearly failed this test, showing a final API content of only 78.0% of the declared amount, far outside of the 90–110% range specified by the pharmacopoeias. Furthermore, it showed a 21% loss of its API content over 6 months at 40°C, grossly exceeding the permitted 5% change. The preparation by Ningbo was labeled for storage “below 25°C.” Clearly, both investigated batches failed accelerated stability testing at 40°C (final API content 69.5% and 74.6%, respectively; Figures 2 and 3 and Supplemental Figure S1) and, by WHO guidelines, therefore had to be tested at “intermediate conditions” of 30°C. After 6 months at 30°C, the API content of batch 160802 was found to be 86.5% of the declared amount and was therefore out of specification, even though the change of the API content was 3% and therefore within the permitted limit of 5%. Batch 160183 showed an API content of 90.7% after 6 months at 30°C and therefore remained in specifications.

The different stability of the investigated preparations under temperature stress conditions is clearly visible in Figure 3, which depicts the time course of the loss of the API content over 6 months at 40°C. This temperature is a more extreme condition than recommended for stability testing of preparations labeled for storage at 2–8°C; therefore, the data depicted in Figure 3 are not directly relevant for pass/fail decisions for most of the preparations, but are still interesting in terms of the stabilizing effect of certain excipients. The most striking observation from these data is that the highest stability of oxytocin was shown by the two products which contained 5 mg/mL of the bacteriostatic agent chlorobutanol. However, in clear contrast to the other investigated products, these two preparations showed marked changes of their pH values, reaching a final value of 2.8 in case of the Sterop product and 3.0 in case of the Ciron product (Supplemental Table S2).

As mentioned earlier, for three of the investigated oxytocin brands, two different batches had been collected, with different manufacturing and expiry dates. Supplemental Figure S1 shows the results of the stability testing for these additional three batches, depicted in the same way as in Figures 2 and 3. Notably, API losses measured for the two different batches of the same brand were very similar in all three cases, indicating that the observed differences in the stability of different brands were primarily because of differences in manufacturing and pharmaceutical formulation, not to differences in age or in pre-sampling storage conditions of the investigated products. Individual measurements for all investigated batches are shown in Supplemental Tables S1 and S2.

### Thermal forced degradation studies of oxytocin.

**Figure 4.**
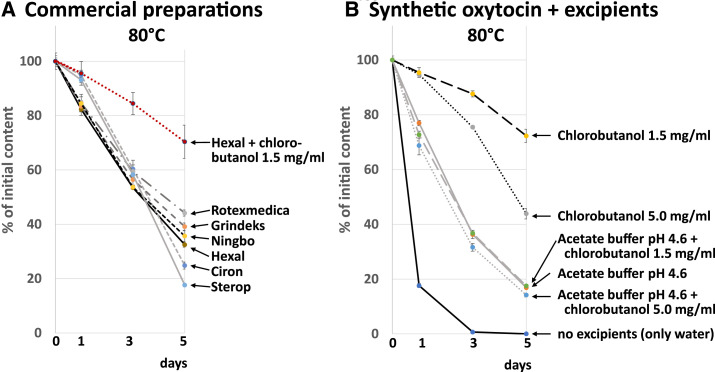
(**A**) Forced thermal degradation studies of commercial oxytocin formulations. Details of the investigated preparations are given in Table 2 and in Supplemental Tables S4 and S5. (**B**) Forced thermal degradation studies of solutions of oxytocin (Sigma-Aldrich/Merck; 10 IU/mL) in the presence of different excipients. This figure appears in color at www.ajtmh.org.

The markedly different stability of the investigated preparations in accelerated stability testing (Figures 2 and 3) stimulated our interest to additionally conduct a forced degradation study at higher temperature, that is, at 80°C for 5 days.^30^ For two of the preparations (Umedica and Biologici; Table 2), the number of remaining vials was insufficient for this experiment, but for the other six brands, the results are shown in Figure 4A. In remarkable similarity to the results at 40°C (Figure 3), the four preparations by Rotexmedica, Grindeks, Ningbo, and Hexal showed decreasing stability in that order, with API losses between 55.9% and 67.5% within 5 days. However, in sharp contrast to the results obtained at 40°C, the highest losses in the API content after 5 days at 80°C were shown by the two preparations by Ciron and Sterop, that is, the preparations containing 5 mg/mL chlorobutanol (API losses of 75.2% and 82.4%, respectively). Conversely, after 1 day, at 80°C, these two preparations still had clearly higher API contents than the other four (Figure 4A).

Chlorobutanol is known to undergo hydrolysis under elevated temperature, forming hydrochloric acid and other acidic reaction products.^40^ This is likely to explain the change of pH of the preparations containing 5 mg/mL chlorobutanol which had been observed already at 40°C (Supplemental Table S2). As may be expected, this effect was even more pronounced at 80°C, with both preparations showing a pH of 2.0 after 5 days, far out of the stability optimum of oxytocin at pH 4.5.^14^

To further investigate the effect of excipients on oxytocin stability, pure synthetic oxytocin in the solid form was purchased from Sigma-Aldrich/Merck and dissolved to a concentration of 10 IU/mL in water, with and without the addition of acetate buffer pH 4.6 (200 mM, i.e., as approximately isotonic solution) and/or chlorobutanol in concentrations of 1.5 mg/mL (8.5 mM) or 5 mg/mL (28 mM). These solutions were subjected to forced degradation at 80°C, and the result is shown in Figure 4B. In pure water, oxytocin degraded completely, falling below the limit of detection within 5 days. This degradation is much more rapid than that observed for any of the investigated commercial preparations (Figure 4A), and that is a further indication that all commercial preparations may have contained buffering or stabilizing agents, even if not declared on the label. Inclusion of acetate buffer had a stabilizing effect on oxytocin solution (83.1% API loss within 5 days). Notably, inclusion of 1.5 mg/mL chlorobutanol had an even stronger stabilizing effect: only 27.7% of the API amount was lost over 5 days at 80°C; the final pH value was determined as 2.8. Inclusion of chlorobutanol in a concentration of 5 mg/mL was less effective for stabilization (56.1% API loss), and, in this case, the final pH value was found to be 2.2, that is, more unfavorable for oxytocin stability.

The stabilizing effects of acetate buffer and chlorobutanol were not additive: in acetate buffer without chlorobutanol, oxytocin degraded with similar velocity as in acetate buffer with added chlorobutanol (1.5 or 5 mg/mL). In the latter experiments, the pH values of the solutions after 5 days at 80°C were 4.6 and 4.5, respectively, indicating that the buffer capacity of 200 mM acetate buffer pH 4.6 was sufficient to compensate for the effect of any acidic hydrolysis products of chlorobutanol.

In a final experiment, chlorobutanol was added to the commercial oxytocin preparation of Hexal, and stability was investigated for 5 days at 80°C. As shown in Figure 4A, addition of 1.5 mg/mL greatly increased the stability of oxytocin under these conditions (29.6% API loss, compared with 67.5% in the absence of chlorobutanol; final pH 2.8). The addition of 5 mg/mL chlorobutanol to the Hexal preparation was less effective for stabilization (API loss 68.4%; Supplemental Table S4), consistent with an observed drastic change of the pH value (final pH 2.0; Supplemental Table S5). These results are similar to the ones observed with the synthetic oxytocin from Sigma-Aldrich/Merck (Figure 4B).

**Figure 5.**
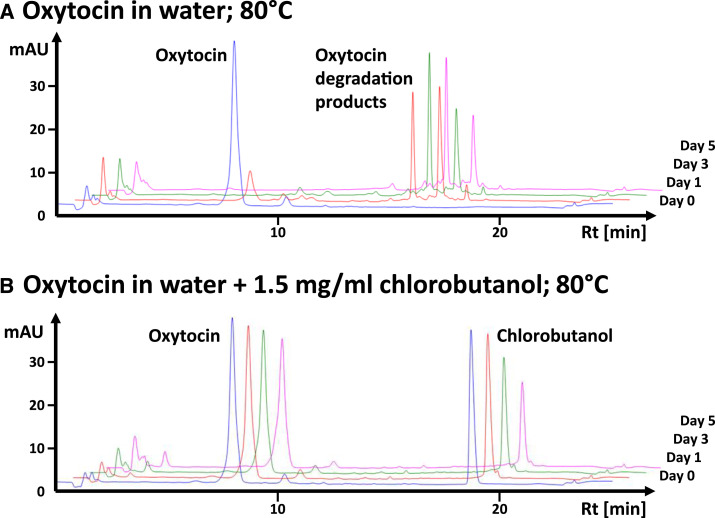
High-performance liquid chromatography chromatograms of oxytocin (Sigma-Aldrich/Merck; 10 IU/mL) in water under forced degradation at 80°C, in the absence and presence of chlorobutanol 1.5 mg/mL. This figure appears in color at www.ajtmh.org.

The HPLC analyses of the samples taken during forced degradation studies showed not only a quantitative effect of chlorobutanol on the velocity of oxytocin degradation but also a striking qualitative effect (Figure 5). In the absence of chlorobutanol, concomitantly to the decrease in the oxytocin peak (retention time 8.2 minutes), several peaks of degradation products with higher retention time (15.1–19.3 minutes) appeared (Figure 5A). This is similar to the HPLC observations of Avanti et al.,^39^ who identified these degradation products as oxytocin trisulfides and tetrasulfides, and as various disulfide-linked oxytocin dimers, some of these showing deamidation of one or more of three amidated carboxyl groups of oxytocin (Figure 1). Notably, in the present study, these decomposition products were not observed in water containing 1.5 mg/mL chlorobutanol (Figure 5B), and the same observation was made for 5 mg/mL chlorobutanol, as well as for the preparations of Ciron and Sterop which each contained 5 mg/mL chlorobutanol. By contrast, all investigated solutions not containing chlorobutanol showed degradation products in the 15.1- to 19.3-minute range.

Neither in the accelerated stability studies nor in the forced degradation studies, visible quality defects such as particles, precipitations, or color changes were observed in any of the investigated products.

## DISCUSSION

An encouraging result from the present study is that six of the eight investigated oxytocin brands passed the accelerated stability testing conducted according to the WHO/ICH.^22,28^ This is even more encouraging as the products were not purchased ex-factory, but from the supply chains in Malawi and Rwanda (and in case of the Hexal product in Germany). Therefore, the extended storage before testing, possibly even at less than optimal storage conditions, had not affected the quality and stability of these products in regard to the tested criteria.

However, two other samples were found not to comply with specifications after accelerated stability testing, and these were the two brands labeled for non-refrigerated storage. The Umedica product carried a storage recommendation “not exceeding 30°C” and therefore had to be tested at 40°C. It clearly failed at this temperature (Figures 2 and 3), and only complied with the stability testing at 30°C, which is appropriate for products labeled for refrigerated storage. By contrast, batch 160802 of the Ningbo product even failed assay testing after 6 months at 30°C, and at 40°C, it showed the poorest result of all eight tested brands. The initial API content (at month 0) of this Ningbo batch was 89.0% of the declared amount. Although it may be argued that the low initial content could have been caused by inappropriate storage before sample collection, it is noteworthy that also two oxytocin samples stating Ningbo as the manufacturer name had been investigated in a survey conducted by the WHO, and both samples failed assay testing because of the insufficient API content.^11^ In the present study, a second batch of Ningbo showed an initial API content of 94.9% of the stated amount. It also failed testing at 40°C, although it passed the test at 30°C.

While this study was in progress, Nguyen et al.^17^ published an investigation of the stability of five oxytocin products purchased ex-factory. Three of these products, labeled for storage at 2–8°C, have also been investigated in the present study, that is, the products by Grindeks, Biologici, and Rotexmedica. The results obtained by Nguyen et al. after 1, 2, and 3 months of storage at 30°C and 40°C are in excellent agreement with those of our study. Nguyen et al. did not test for the full period of 6 months; therefore, results at that time point cannot be compared, but there is no evidence pointing at any relevant differences. Nguyen et al. also investigated one European oxytocin brand labeled for storage at ≤ 25°C, as well as one Argentinian product which had formerly been labeled for storage at ≤ 25°C but had been relabeled for refrigerated storage just before their study. They reported that, at 30°C and 40°C, these two preparations exhibited very similar stability profiles as the three products labeled for refrigerated storage. Only after 4 months at 40°C, and especially in a forced degradation study at 50°C, the European product labeled for storage at ≤ 25°C showed much higher API losses than the others, concomitant with a sharp decrease in the pH value. That preparation contained 5 mg/mL chlorobutanol. The authors concluded that, of the products tested, those designated for storage at ≤ 25°C provided no stability benefit over those labeled for refrigerated storage.

The present study investigated six oxytocin brands labeled for storage at 2–8°C, as well as two brands labeled for storage at ≤ 25°C and at ≤ 30°C. The two latter products had been manufactured in China and India, respectively. The data of the present study are therefore complementary to the data provided by Nguyen et al. in regard to the range of tested products. Our study results give further support to the conclusion that products designated for storage at ≤ 25°C do not provide stability benefits over those labeled for refrigerated storage. In addition, our study shows that some of the former products may even fail the relevant WHO/ICH specifications for the stability of finished pharmaceutical products.

Five of the eight oxytocin brands investigated in this study passed stability testing with excellent results, that is, with final API contents not more than 2.6% below the amount stated on the label after 6 months at 30°C (Supplemental Table S1). Notably, all these five brands were WHO prequalified and/or produced in countries with SRAs. By contrast, of the three preparations produced in India and China, that is, in countries without SRAs, two showed noncompliance with pharmacopoeial specifications after accelerated stability testing, and one passed testing with just borderline results. These poor stability outcomes must not be generalized to all manufacturers and medicines from non-SRA countries. There are certainly manufacturers (usually larger companies), for example, in India and China, whose products are as good as generic medicines produced in countries with SRAs.^41^ However, there are also companies (usually smaller ones) in these two countries whose products show a large rate of substandard medicines.^42^ It should be both an ethical and an economical interest of the authorities in India and China to address and eliminate this problem.

For the brands which were WHO prequalified and/or produced in countries with SRAs, excipients were correctly declared. By contrast, for the three preparations without WHO prequalification and produced in countries without SRAs, the excipients were not declared correctly, which is a violation of registration requirements in most countries.

The results of the present study strongly support the maxim “Buy Quality Oxytocin, Keep It Cool” which is advocated by the Reproductive Health Supplies Coalition and other international stakeholders.^21,43–46^ Given the lack of consistent evidence that products labeled for non-refrigerated storage show better temperature stability than those labeled for storage at 2–8°C, and furthermore given the clear evidence that manufacturers’ storage recommendations of “below 25°C” and even of “below 30°C” cannot be reliably complied with in many facilities in LMICs,^13^ procurement of oxytocin injections should be limited to brands labeled for refrigerated storage, and national medicines regulatory agencies should consider to only register oxytocin products with this storage recommendation.^43,44^ This will also help to avoid confusion of the personnel in supply chains and health facilities by different storage recommendations for different oxytocin brands.^13,18,47,48^ All efforts should be made to maintain the cold chain for oxytocin from the manufacturer up to the patient. The WHO/UNICEF recommendation to allow storage of oxytocin in the ubiquitous cold chain facilities of the Expanded Program on Immunization should be implemented as widely as possible for this purpose.^49^

Given the current shortcomings of the cold chain facilities in LMICs, however, it may still not be possible to avoid short-term exposures of oxytocin to ambient temperatures. In this context, the data depicted in Figure 2 may be of interest, showing that all products labeled for storage at 2–8°C were remarkably stable at 25°C in our stability study. Even at 30°C, only very moderate losses of the API content were observed over the investigated period. This indicates that accidental storage of such oxytocin preparations outside of the cold chain for limited time periods may not necessitate the immediate disposal of the products, and this is consistent with the information given, for example, in the leaflet of the Grindeks product (Table 2), stating that it “may be stored up to 30°C for 3 months, but must then be discarded.”

According to Hawe et al.,^14^ degradation of oxytocin in a 10-IU/mL solution of pH 4.5 has an activation energy of 128 ± 3.8 kJ/mol and follows (pseudo-) first-order kinetics. Using the Arrhenius equation,^14^ we calculated that a temperature increase just from 25°C to 30°C would increase the degradation reaction rate by a factor of 2.3. Thakral et al.^21^ extrapolated rate constants for oxytocin degradation at 25–40°C from experimentally obtained rate constants at higher temperatures, under the assumption that the mechanism of reaction remains unchanged. Thereby, they calculated the time required for a 10% loss of oxytocin content at temperatures of 25°C, 30°C, and 40°C as 183 days, 82 days, and 17 days, respectively, at pH 4.5. Figures 2 and 3 of the present study clearly show that the degradation rates observed experimentally at these temperatures for commercial oxytocin products were much lower, indicating different reaction mechanisms at lower temperatures and/or the presence of stabilizing agents.

Attempts to devise more heat-stable formulations of oxytocin have been described extensively in the literature,^14,36–39,50^ mostly focusing on the inclusion of certain buffers and divalent metal ions. However, to the best of our knowledge, the striking effect of chlorobutanol, a bacteriostatic agent widely used as a preservative in pharmaceuticals,^40^ both on the velocity on oxytocin degradation (Figure 4) and on the type of degradation products formed (Figure 5) has not been described in the literature in any detail up to now. This stabilizing effect has only been very briefly mentioned in an abstract by Liu et al.,^51^ and a molecular dynamics computer simulation by Xu et al.^52,53^ suggested that chlorobutanol can reduce the number of hydrogen bonds between oxytocin and water and prevent oxytocin molecules from aggregating. Evidence has been presented that divalent metal ions may lead to a change of the conformation of oxytocin in certain buffers, thereby shielding the intramolecular disulfide bond (Figure 1) and diminishing degradation reactions which involve that site of the molecule.^36–39^ It is tempting to speculate that chlorobutanol may have a similar effect on oxytocin. Possibly, 200 mM acetate buffer may prevent this conformational change, explaining the lack of a stabilizing effect of chlorobutanol observed in this buffer (Figure 4B).

Chlorobutanol itself degrades at higher temperatures, as clearly visible in Figure 5 which shows 47.5% degradation of chlorobutanol in 5 days at 80°C under the used conditions. The degradation leads to acidic degradation products^40^ and lowers the pH value, which then may strongly deviate from the oxytocin stability optimum (pH 4.5), especially when a high concentration of chlorobutanol (i.e., 5 mg/mL) is used in weakly buffered solutions. The detrimental effect of the progressive lowering of the pH value would then compete with the stabilizing effect of chlorobutanol on oxytocin, and this may offer a plausible explanation for the observation depicted in Figure 4A (Ciron and Sterop products) that chlorobutanol 5 mg/mL first increased and later on lowered oxytocin stability at 80°C. The lowering of the pH and the resulting decrease in oxytocin stability was also observed in the forced degradation experiment by Nguyen et al.,^17^ similar to our results. This effect may, however, not be relevant at actual storage temperatures of oxytocin because the hydrolysis of chlorobutanol only occurs at higher temperatures.

It may well be worthwhile to investigate the mechanism of the stabilization of oxytocin by chlorobutanol using appropriate methods, especially liquid chromatography-mass spectrometry (LC-MS) analysis for the identification of the degradation products^39^ and nuclear magnetic resonance (NMR) spectroscopy to investigate conformational changes of oxytocin.^36^ However, such investigations exceed the scope of the present study.

A heat-stable formulation of carbetocin,^54^ an oxytocin analogue which is already widely used for the prevention of PPH after caesarean delivery, has very recently been added to the WHO Essential Medicines List for use in PPH.^1^ If the announcement of its manufacturer is put into practice that the price of this product will be made comparable to that of oxytocin in LMICs,^55^ then this medicine may become a further valuable option to ensure good-quality medication against PPH also in facilities where storage at 2–8°C cannot be ensured.

### Limitations of this study.

As mentioned earlier, in this study, oxytocin samples were investigated which had been purchased from wholesalers and medical stores in Malawi and Rwanda, that is, preparations which reflect the quality and stability of medicines circulating in these countries. It has to be considered that these samples had already been stored for extended periods after manufacturing, and that the conditions of this storage are not known. Samples were not purchased from retail outlets to minimize the influence of varying conditions during prior storage on the study results. Although the observed differences in the stability of the investigated samples are valid and important, the data provided here do not provide definitive proof that certain products did not comply with their stability requirements already at the time of manufacture.

## Supplemental figure and tables

Supplemental materials
