# Supplementary material for: Stability of Oxytocin Preparations in Malawi and Rwanda: Stabilizing Effect of Chlorobutanol
Source: Am J Trop Med Hyg. 2020 Aug 3;103(5):2129–41. doi: 10.4269/ajtmh.20-0255 (PMC7646793; doi:10.4269/ajtmh.20-0255)
Supplement: Supplementary file 1 [file tpmd200255.SD1.pdf]

SUPPLEMENTARY INFORMATION

Figure S1: Accelerated stability testing of commercial oxytocin preparations: results of additional batches

Oxytocin content after 6 months at different temperatures:

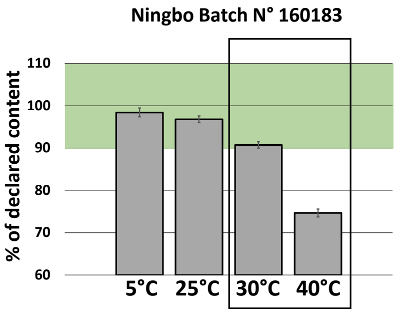

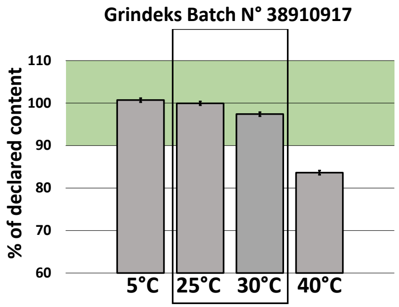

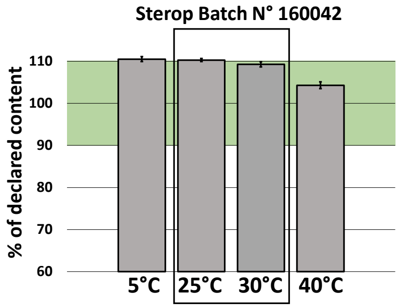

Change of oxytocin content over 6 months at 40°C:

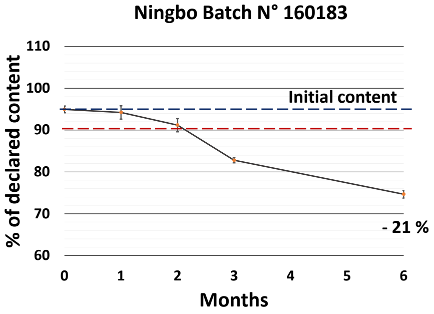

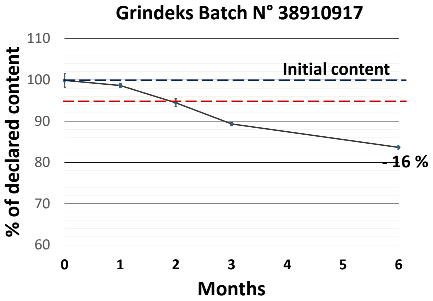

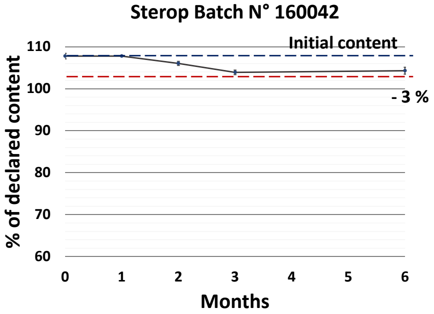

Table S1: Accelerated stability testing of commercial oxytocin preparations: change of oxytocin content over 6 months at different temperatures

		Oxytocin mean content (% of declared content)									
**Condition**	**Sample**	**Month 0**	**RSD**	**Month 1**	**RSD**	**Month 2**	**RSD**	**Month 3**	**RSD**	**Month 6**	**RSD**
**Accelerated Ambient ICH 40 +/- 2° 75% RH +/- 5°**	**Ningbo 160802**	89.0	1.41%	86.1	0.90%	85.3	0.48%	77.5	1.65%	69.5	1.05%
	**Ningbo 160183**	94.9	0.87%	94.2	1.69%	91.2	1.74%	82.8	0.76%	74.6	1.23%
	**Umedica**	98.1	1.29%	96.9	0.28%	93.0	1.06%	84.2	0.30%	78.0	0.70%
	**Ciron**	92.7	1.69%	94.4	0.70%	86.9	2.10%	86.0	1.34%	84.2	1.38%
	**Biologici**	99.5	0.53%	100.4	0.22%	97.8	1.15%	90.7	0.26%	85.5	0.54%
	**Sterop 160269**	99.6	1.11%	97.3	0.77%	97.8	0.19%	95.2	1.01%	95.4	0.80%
	**Sterop 160042**	107.8	0.62%	107.8	0.12%	106.1	0.45%	103.9	0.52%	104.3	0.77%
	**Rotexmedica**	102.4	0.89%	102.3	0.80%	99.8	0.53%	93.5	1.55%	89.6	0.29%
	**AS Grindeks 37711116**	100.9	0.82%	100.7	1.37%	96.1	0.66%	90.4	0.83%	85.2	0.42%
	**AS Grindeks 38910917**	99.9	1.65%	98.7	0.54%	94.5	0.99%	89.3	0.47%	83.6	0.59%
	**Hexal**	101.2	0.15%	98.4	0.69%	94.2	0.05%	88.2	0.82%	77.3	0.46%
**Zone IVa; Accelerated Refrigerated ICH more severe conditions 30° +/- 2° 65% RH +/- 5%**	**Ningbo 160802**	89.0	1.41%	90.3	0.87%	87.5	0.43%	86.1	0.64%	86.5	0.60%
	**Ningbo 160183**	94.9	0.87%	97.5	1.29%	98.0	1.78%	93.6	1.23%	90.7	0.80%
	**Umedica**	98.1	1.29%	100.0	0.47%	99.1	0.22%	93.1	0.39%	94.3	1.45%
	**Ciron**	92.7	1.69%	94.1	0.52%	92.5	1.70%	92.5	0.63%	89.3	1.55%
	**Biologici**	99.5	0.53%	102.4	0.38%	102.2	0.96%	95.8	0.52%	97.6	0.81%
	**Sterop 160269**	99.6	1.11%	99.6	0.11%	98.8	0.03%	97.0	0.21%	101.3	0.95%
	**Sterop 160042**	107.8	0.62%	108.7	0.32%	108.0	0.48%	104.9	0.08%	109.3	0.55%
	**Rotexmedica**	102.4	0.89%	104.2	0.16%	103.8	0.74%	99.6	0.45%	100.5	0.64%
	**AS Grindeks 37711116**	100.9	0.82%	101.9	0.57%	101.4	0.65%	97.8	0.89%	98.6	0.79%
	**AS Grindeks 38910917**	99.9	1.65%	101.0	0.54%	99.3	0.47%	95.6	0.46%	97.4	0.48%
	**Hexal**	101.2	0.15%	101.2	0.70%	100.5	0.40%	98.3	0.82%	98.4	0.38%

**Zone II; Accelerated Refrigerated ICH 25° +/- 2° 60% RH +/- 5%**	**Ningbo 160802**	89.0	1.41%	88.4	0.37%	90.2	1.06%	86.0	1.52%	88.2	0.44%
	**Ningbo 160183**	94.9	0.87%	100.2	1.35%	97.7	1.47%	96.9	0.75%	96.8	0.84%
	**Umedica**	98.1	1.29%	101.1	0.37%	100.3	0.37%	95.1	0.44%	97.9	1.40%
	**Ciron**	92.7	1.69%	94.3	1.63%	95.1	1.17%	92.8	1.20%	94.5	0.93%
	**Biologici**	99.5	0.53%	102.7	0.46%	102.8	0.96%	98.0	0.20%	100.5	0.48%
	**Sterop 160269**	99.6	1.11%	100.0	0.37%	98.5	0.33%	97.4	0.86%	103.5	0.35%
	**Sterop 160042**	107.8	0.62%	107.4	0.28%	108.1	0.39%	105.4	0.33%	110.3	0.32%
	**Rotexmedica**	102.4	0.89%	104.4	0.56%	104.0	0.69%	100.4	0.98%	102.6	0.83%
	**AS Grindeks 37711116**	100.9	0.82%	102.3	0.27%	102.0	0.93%	98.7	1.19%	100.7	0.99%
	**AS Grindeks 38910917**	99.9	1.65%	101.1	0.30%	100.0	1.24%	96.6	0.85%	99.9	0.44%
	**Hexal**	101.2	0.15%	101.7	0.87%	101.3	0.35%	99.5	0.90%	101.1	0.90%

**Control long term testing conditions, refrigerated 5° +/- 3°**	**Ningbo 160802**	89.0	1.41%	88.8	0.17%	90.9	1.12%	90.2	0.10%	91.6	1.45%
	**Ningbo 160183**	94.9	0.87%	97.4	0.08%	97.5	1.50%	96.0	1.67%	98.4	1.06%
	**Umedica**	98.1	1.29%	100.5	0.63%	100.7	0.39%	95.7	0.92%	99.5	0.65%
	**Ciron**	92.7	1.69%	97.5	0.74%	94.6	1.66%	94.4	0.40%	95.3	1.44%
	**Biologici**	99.5	0.53%	102.3	0.76%	103.6	0.16%	97.4	1.29%	100.3	0.91%
	**Sterop 160269**	99.6	1.11%	100.0	0.25%	99.4	0.42%	98.1	0.48%	103.6	0.46%
	**Sterop 160042**	107.8	0.62%	109.1	0.20%	108.5	0.29%	105.6	0.48%	110.5	0.53%
	**Rotexmedica**	102.4	0.89%	104.7	0.46%	105.5	0.83%	100.8	0.19%	103.5	0.87%
	**AS Grindeks 37711116**	100.9	0.82%	102.3	0.55%	103.4	0.75%	99.7	0.77%	102.5	1.06%
	**AS Grindeks 38910917**	99.9	1.65%	101.1	0.39%	101.6	0.45%	99.2	1.02%	100.7	0.48%
	**Hexal**	101.2	0.15%	101.9	0.92%	102.4	0.67%	100.4	0.55%	103.6	0.55%

RSD: relative standard deviation. ICH: International Conference on Harmonization. RH: relative humidity. Oxytocin content calculated as the mean of the results of three individual vials per sample.

Table S2: Accelerated stability testing of commercial oxytocin preparations: change of pH value over 6 months at different temperatures

		Oxytocin mean pH-values									
Condition	Sample	Month 0	RSD	Month 1	RSD	Month 2	RSD	Month 3	RSD	Month 6	RSD
Accelerated Ambient ICH 40 +/- 2° 75% RH +/- 5°	Ningbo 160802	3.9	0.63%	3.9	1.33%	4.3	1.28%	3.9	0.82%	4.2	1.04%
	Ningbo 160183	4.1	0.88%	4.0	0.37%	4.4	1.10%	4.0	0.65%	4.3	0.57%
	Umedica	4.2	1.24%	4.1	0.40%	4.5	1.62%	4.1	0.30%	4.3	0.74%
	Ciron	4.5	2.38%	3.5	1.69%	3.7	2.94%	3.1	1.16%	3.0	2.18%
	Biologici	3.8	0.95%	3.8	0.46%	4.1	2.29%	3.8	0.99%	4.0	0.45%
	Sterop 160269	4.0	0.57%	3.3	1.19%	3.5	2.82%	3.0	1.49%	2.8	1.35%
	Sterop 160042	3.8	0.22%	3.3	1.02%	3.5	2.37%	2.9	1.28%	2.8	1.45%
	Rotexmedica	4.0	0.47%	3.9	0.47%	4.3	1.90%	3.9	0.31%	4.1	0.30%
	AS Grindeks 37711116	4.1	0.64%	4.0	0.44%	4.4	1.93%	4.0	0.32%	4.2	0.32%
	AS Grindeks 38910917	4.1	0.46%	4.0	0.33%	4.4	1.69%	4.0	0.29%	4.2	0.33%
	Hexal	4.2	0.13%	4.1	1.20%	4.6	1.75%	4.3	0.46%	4.5	1.48%
Zone IVa; Accelerated Refrigerated ICH more severe conditions 30° +/- 2° 65% RH +/- 5%	Ningbo 160802	3.9	0.63%	3.9	0.77%	4.3	1.58%	3.9	1.15%	4.1	0.50%
	Ningbo 160183	4.1	0.88%	4.0	0.62%	4.4	1.42%	4.0	0.52%	4.2	0.38%
	Umedica	4.2	1.24%	4.1	0.43%	4.5	1.46%	4.1	1.79%	4.3	0.32%
	Ciron	4.5	2.38%	4.0	0.89%	4.4	1.74%	3.9	1.38%	3.8	1.45%
	Biologici	3.8	0.95%	3.8	0.47%	4.2	1.52%	3.8	0.57%	4.0	0.38%
	Sterop 160269	4.0	0.57%	3.7	0.79%	4.0	1.58%	3.6	0.63%	3.5	0.67%
	Sterop 160042	3.8	0.22%	3.6	0.79%	3.9	1.50%	3.4	0.75%	3.5	0.76%
	Rotexmedica	4.0	0.47%	3.9	0.85%	4.3	1.40%	3.9	0.44%	4.1	0.36%
	AS Grindeks 37711116	4.1	0.64%	4.0	0.53%	4.4	1.24%	4.0	0.37%	4.2	0.28%
	AS Grindeks 38910917	4.1	0.46%	4.0	0.56%	4.4	1.22%	4.0	0.41%	4.2	0.29%
	Hexal	4.2	0.13%	4.1	1.11%	4.6	1.39%	4.2	0.36%	4.4	0.69%

**Zone II; Accelerated Refrigerated ICH 25° +/ 2° 60% RH +/ 5%**	**Ningbo 160802**	3.9	0.63%	3.9	0.54%	4.3	1.28%	3.9	1.03%	4.1	0.43%
	**Ningbo 160183**	4.1	0.88%	4.0	0.66%	4.4	1.95%	4.0	0.56%	4.2	0.85%
	**Umedica**	4.2	1.24%	4.2	1.67%	4.5	1.61%	4.1	0.47%	4.3	0.60%
	**Ciron**	4.5	2.38%	4.2	3.03%	4.5	2.22%	4.1	0.53%	4.3	5.76%
	**Biologici**	3.8	0.95%	3.8	0.74%	4.2	2.07%	3.8	0.49%	4.0	0.57%
	**Sterop 160269**	4.0	0.57%	3.8	0.80%	4.2	1.24%	3.8	0.55%	3.8	0.65%
	**Sterop 160042**	3.8	0.22%	3.6	1.11%	4.0	1.55%	3.6	0.87%	3.7	0.58%
	**Rotexmedica**	4.0	0.47%	3.9	0.70%	4.3	1.54%	3.9	0.25%	4.1	0.30%
	**AS Grindeks 37711116**	4.1	0.64%	4.0	0.48%	4.4	1.57%	4.0	0.26%	4.2	0.67%
	**AS Grindeks 38910917**	4.1	0.46%	4.0	0.46%	4.4	1.49%	4.0	0.26%	4.2	0.29%
	**Hexal**	4.2	0.13%	4.0	1.70%	4.6	1.59%	4.1	0.58%	4.4	0.40%
**Control long term testing conditions, refrigerated 5° +/ 3°**	**Ningbo 160802**	3.9	0.63%	3.9	1.10%	4.3	1.99%	3.9	1.52%	4.1	0.81%
	**Ningbo 160183**	4.1	0.88%	4.1	0.36%	4.4	1.13%	4.0	0.54%	4.2	0.74%
	**Umedica**	4.2	1.24%	4.1	0.71%	4.5	1.09%	4.1	0.33%	4.3	0.34%
	**Ciron**	4.5	2.38%	4.2	1.40%	4.8	3.84%	4.2	0.24%	4.4	1.28%
	**Biologici**	3.8	0.95%	3.8	0.47%	4.2	1.73%	3.8	0.57%	4.0	0.55%
	**Sterop 160269**	4.0	0.57%	3.8	1.01%	4.3	1.49%	3.9	0.88%	4.0	0.44%
	**Sterop 160042**	3.8	0.22%	3.7	0.40%	4.1	1.76%	3.7	0.66%	3.9	0.56%
	**Rotexmedica**	4.0	0.47%	3.9	0.52%	4.3	1.64%	3.9	0.26%	4.1	0.39%
	**AS Grindeks 37711116**	4.1	0.64%	4.0	0.37%	4.4	1.42%	4.0	0.46%	4.2	0.42%
	**AS Grindeks 38910917**	4.1	0.46%	4.0	0.42%	4.5	1.64%	4.0	0.41%	4.2	0.43%
	**Hexal**	4.2	0.13%	4.1	0.24%	4.5	1.60%	4.1	0.59%	4.3	0.59%

RSD: relative standard deviation. ICH: International Conference on Harmonization. RH: relative humidity. pH values calculated as the mean of the results of three individual vials per sample, each vial tested twice, yielding 6 measurements per sample.

Table S3: Determination of intermediate precision[1] of oxytocin assay, using data from five-point calibration curves

	Mean AUC (mAU*s) Month 0	*RSD*	Mean AUC (mAU*s) Month 1	*RSD*	Mean AUC (mAU*s) Month 2	*RSD*	Mean AUC (mAU*s) Month 3	*RSD*	Mean AUC (mAU*s) Month 6	*RSD*	**Mean AUC (mAU*s) all months**	**RSD**
USP Reference 11.5 IU/ml	795.37	*1.25%*	809.72	*0.09%*	818.59	*0.43%*	807.35	*0.23%*	770.90	*0.62%*	**800.39**	**2.31%**
USP Reference 10 IU/ml	697.92	*1.31%*	699.32	*0.24%*	708.83	*0.37%*	699.52	*0.29%*	670.62	*0.79%*	**695.24**	**2.08%**
USP Reference 7 IU/ml	478.30	*0.67%*	483.76	*0.86%*	495.49	*0.08%*	485.75	*1.02%*	468.38	*0.87%*	**482.33**	**2.07%**
USP Reference 5 IU/ml	333.61	*1.52%*	342.19	*0.28%*	344.07	*0.24%*	344.91	*1.73%*	322.33	*1.19%*	**337.42**	**2.83%**
USP Reference 2 IU/ml	123.83	*3.24%*	131.64	*0.92%*	130.95	*1.01%*	140.46	*2.71%*	120.89	*1.10%*	**129.56**	**5.89%**

AUC: area under the curve. RSD: relative standard deviation. IU: international units. Each mean AUC value was calculated from on three HPLC measurements, but each mean AUC value of USP Reference 10 IU/ml from five HPLC measurements.

[1]**Reference:** ICH, 2005. Validation of analytical procedures: Text and methodology Q2(R1). Available from: https://database.ich.org/sites/default/files/Q2_R1__Guideline.pdf.

Table S4: Forced thermal degradation studies of solutions of oxytocin (Sigma-Aldrich/Merck; 10 IU/ml) in the presence of different excipients, and of commercial oxytocin formulations: change of oxytocin content over 5 days at 80°C

	Oxytocin content (% of initial content)							
	Day 0	*RSD*	Day 1	*RSD*	Day 3	*RSD*	Day 5	*RSD*
**10 IU/ml synthetic oxytocin in destilled water**	100.0	*0.44%*	17.6	*4.00%*	0.7	*16.98%*	0.0	*0.00%*
**10 IU/ml synthetic oxytocin in sodium acetate buffer pH 4.6**	100.0	*0.04%*	77.0	*1.25%*	36.3	*4.21%*	16.9	*1.29%*
**10 IU/ml synthetic oxytocin in destilled water containing 5mg/ml chlorobutanol**	100.0	*0.00%*	94.7	*0.34%*	75.5	*0.72%*	43.9	*4.36%*
**10 IU/ml synthetic oxytocin in destilled water containing 1.5mg/ml chlorobutanol**	100.0	*0.75%*	95.4	*1.89%*	87.6	*1.27%*	72.3	*3.34%*
**10 IU/ml synthetic oxytocin in sodium acetate buffer pH 4.6 containing 5mg/ml chlorobutanol**	100.0	*0.36%*	68.7	*4.93%*	31.6	*4.45%*	14.2	*4.90%*
**10 IU/ml synthetic oxytocin in sodium acetate buffer pH 4.6 containing 1.5mg/ml chlorobutanol**	100.0	*1.46%*	72.7	*1.75%*	36.7	*1.47%*	17.5	*2.79%*
**Hexal (batch HWZ7694[a])**	100.0	*0.04%*	82.3	*2.72%*	53.5	*0.98%*	32.5	*2.86%*
**Hexal (batch HWZ7694 [a]) containing 5mg/ml chlorobutanol**	100.0	*0.76%*	93.9	*2.23%*	64.8	*6.51%*	31.6	*11.46%*
**Hexal (batch HWZ7694 [a]) containing 1.5mg/ml chlorobutanol**	100.0	*2.01%*	95.6	*4.57%*	84.4	*4.83%*	70.4	*8.68%*
**Ningbo 160183**	100.0	*3.07%*	84.5	*2.91%*	53.7	*1.37%*	35.7	*5.51%*
**Ciron**	100.0	*0.16%*	94.9	*1.89%*	60.3	*5.32%*	24.8	*5.19%*
**Sterop 160269**	100.0	*0.70%*	93.1	*0.97%*	58.3	*0.31%*	17.6	*1.91%*
**Rotexmedica**	100.0	*0.68%*	84.7	*3.70%*	59.4	*4.10%*	44.1	*2.58%*
**AS Grindeks 38910917**	100.0	*1.22%*	83.4	*2.45%*	56.4	*3.67%*	39.1	*3.47%*

IU: international unit. RSD relative standard deviation.

[a] Batch HWZ7694 had identical packaging information as batch HC0075 listed in Table 2, but expiry date June 2021.

Table S5: Forced thermal degradation studies of solutions of oxytocin (Sigma-Aldrich/Merck; 10 IU/ml) in the presence of different excipients, and of commercial oxytocin formulations: change of pH values over 5 days at 80°C

	**pH values**							
	**Day 0**	***RSD***	**Day 1**	***RSD***	**Day 3**	***RSD***	**Day 5**	***RSD***
**10 IU/ml synthetic oxytocin in destilled water**	NA	*NA*	NA	*NA*	NA	*NA*	NA	*NA*
**10 IU/ml synthetic oxytocin in sodium acetate buffer pH 4.6**	4.7	*0.15%*	NA	*NA*	NA	*NA*	NA	*NA*
**10 IU/ml synthetic oxytocin in destilled water containing 5mg/ml chlorobutanol**	5.6	*2.65%*	2.9	*0.24%*	2.4	*0.30%*	2.2	*0.65%*
**10 IU/ml synthetic oxytocin in destilled water containing 1.5mg/ml chlorobutanol**	6.4	*2.33%*	3.9	*0.18%*	3.1	*0.23%*	2.8	*2.00%*
**10 IU/ml synthetic oxytocin in sodium acetate buffer pH 4.6 containing 5mg/ml chlorobutanol**	4.6	*0.00%*	4.7	*0.30%*	4.5	*0.16%*	4.5	*0.16%*
**10 IU/ml synthetic oxytocin in sodium acetate buffer pH 4.6 containing 1.5mg/ml chlorobutanol**	4.7	*0.60%*	4.6	*0.15%*	4.6	*0.15%*	4.6	*0.30%*
**Hexal (batch HWZ7694 [a])**	4.2	*0.13%*	4.2	*0.50%*	4.6	*0.15%*	4.3	*0.16%*
**Hexal (batch HWZ7694 [a]) containing 5mg/ml chlorobutanol**	4.6	*0.76%*	NA	*NA*	2.3	*0.31%*	2.0	*0.35%*
**Hexal (batch HWZ7694 [a]) containing 1.5mg/ml chlorobutanol**	4.6	*0.77%*	3.8	*0.75%*	2.9	*0.24%*	2.8	*0.25%*
**Ningbo 160183**	4.1	*0.88%*	4.0	*0.18%*	4.4	*1.13%*	4.1	*1.38%*
**Ciron**	4.5	*2.38%*	2.7	*0.26%*	2.6	*1.66%*	2.0	*0.70%*
**Sterop 160269**	4.0	*0.57%*	2.7	*0.53%*	2.5	*1.14%*	2.0	*0.36%*
**Rotexmedica**	4.0	*0.47%*	3.9	*0.00%*	4.2	*1.17%*	3.9	*0.18%*
**AS Grindeks 38910917**	4.1	*0.46%*	4.0	*0.35%*	4.3	*0.82%*	4.0	*0.18%*

IU: international unit. RSD: relative standard deviation. NA: no data available.

[a] Batch HWZ7694 had identical packaging information as batch HC0075 listed in Table 2, but expiry date June 2021.
